# Improved small blob detection in 3D images using jointly constrained deep learning and Hessian analysis

**DOI:** 10.1038/s41598-019-57223-y

**Published:** 2020-01-15

**Authors:** Yanzhe Xu, Teresa Wu, Fei Gao, Jennifer R. Charlton, Kevin M. Bennett

**Affiliations:** 1 School of Computing, Informatics, and Decision Systems Engineering, Arizona State University, 699S Mill Ave, Tempe, AZ 85281 USA; 2 Department of Pediatrics, Division Nephrology, University of Virginia, Charlottesville, VA 22908 USA; 3 Department of Radiology, Washington University, St. Louis, MO 63130 USA

**Keywords:** Image processing, Diagnostic markers, Glomerular diseases

## Abstract

Imaging biomarkers are being rapidly developed for early diagnosis and staging of disease. The development of these biomarkers requires advances in both image acquisition and analysis. Detecting and segmenting objects from images are often the first steps in quantitative measurement of these biomarkers. The challenges of detecting objects in images, particularly small objects known as blobs, include low image resolution, image noise and overlap between the blobs. The Difference of Gaussian (DoG) detector has been used to overcome these challenges in blob detection. However, the DoG detector is susceptible to over-detection and must be refined for robust, reproducible detection in a wide range of medical images. In this research, we propose a joint constraint blob detector from U-Net, a deep learning model, and Hessian analysis, to overcome these problems and identify true blobs from noisy medical images. We evaluate this approach, UH-DoG, using a public 2D fluorescent dataset for cell nucleus detection and a 3D kidney magnetic resonance imaging dataset for glomerulus detection. We then compare this approach to methods in the literature. While comparable to the other four comparing methods on recall, the UH-DoG outperforms them on both precision and F-score.

## Introduction

There is great interest in tailoring diagnostic and therapeutic tools to individual patients. This concept reflects the growing recognition that there is significant variability between individuals. As therapies focus on molecular targets, diagnostic medical imaging tools must reveal focal pathologies and the effects of therapy in each patient. High-resolution object detection and image segmentation are thus critical to obtaining meaningful data in a heterogeneous image.

In image analysis, detection is used to identify objects such as organs and tumors, and segmentation is used to isolate the objects from an image. While large objects can often be automatically or semi-automatically isolated, small objects (blobs) are difficult to detect and segment. Blobs can range in size and location in images. Examples of blobs include cells or cell nuclei in images from optical microscopy^1^, exudative lesions in images of the retina^2^, breast lesions in ultrasound images^3^, and glomeruli in magnetic resonance (MR) images of the kidney^4–6^. Major challenges to detecting these blobs include low image resolution and high image noise. The small blobs are often numerous and can overlap each other. Many approaches have been proposed for blob detection^7–9^ of which intensity thresholding is among the most common^10^. Intensity thresholding assumes that the blobs have consistently different intensities from the background. Global differences can be addressed with a fixed threshold and local differences can be addressed with an adaptive threshold^11,12^. However, the assumptions required for consistent thresholding are often violated, and thresholding alone can lead to erroneous detection or segmentation. To address this, researchers have proposed multi-step pipelines^13,14^ in which thresholding is only the first step. Intensity-based features are then derived using filters for improved detection. One popular class of filters is based on mathematical morphology^15,16^. Operators such as erosion, dilation, opening and closing allow geometrical and topological properties of objects. This approach often begins with selected seed points in the image and iteratively adds connected points to form labeled regions. Mathematical morphology is preferred when the blobs are relatively large in size and small in number. Weaknesses of this approach include the tendency to under-segment and diminished performance in the presence of noise. Under-segmentation occurs when multiple blobs within close proximity are detected as one, resulting in an erroneously low detected number. Another type of filter is based on space transformation. For example, Radial-Symmetry^17^, a point detector for small blobs, uses radially symmetric space as a transformation space to detect radially symmetric blobs. SIFT^18^, SURF^19^ and BRISK^20^ are region detectors. Each of the region detectors extracts scale invariant features to detect small objects but may suffer from poor performance in optical imaging^21^. Recently, the Laplacian of Gaussian (LoG) detector^22,23^, from scale space theory, has attracted attention in blob detection^8,24^. Similar to the radially symmetric detector, the LoG detector is unreliable in detecting rotationally asymmetric blobs. To solve this, LoG extensions have been proposed, including the Difference of Gaussian (DoG)^18,25–27^ and the Generalized Laplacian of Gaussian (gLoG)^21^. While each approach detects small blobs to some extent, non-blob objects are detected as false blob candidates resulting in over-detection. A post-pruning procedure can remove false blob candidates, but results have been inconsistent^28^.

Here we focus on detecting individual glomeruli in MR images of the kidney as a specific blob detection problem. To date, most biomarkers of kidney pathology have come from histology using destructive techniques that estimate glomerular number^29–31^. A non-destructive imaging approach to measuring nephron endowment provides a new marker for renal health and susceptibility to kidney disease. Cationic ferritin enhanced MRI (CFE-MRI) enables the detection of glomeruli in animals^32,33^ and in human kidneys^5,32–34^. Because each glomerulus is associated with a nephron, CFE-MRI may provide an important imaging marker to detect changes in the number of nephrons and susceptibility to renal and cardiovascular disease^5^. Glomerulus detection by CFE-MRI presents difficulties because glomeruli are small and have a spatial frequency similar to image noise. Zhang *et al*. developed the Hessian-based Laplacian of Gaussian (HLoG) detector^1^ and the Hessian-based Difference of Gaussian (HDoG) detector^28^ to automatically detect glomeruli in CFE-MR images. They employed the LoG or DoG to smooth the images, followed by Hessian analysis of each voxel for pre-segmentation. Since LoG and DoG suffer from over-detection, a Variational Bayesian Gaussian Mixture Model (VBGMM) was implemented as a final step. LoG and DoG were the first two detectors applied to MR images of the kidney to identify glomeruli. However, deriving Hessian-based features from each blob candidate is computationally expensive, limiting high-throughput studies. In addition, unsupervised learning using the VBGMM in the post-pruning procedure requires a number of carefully tuned parameters for optimal clustering. Here we propose a new approach, termed UH-DoG, which applies joint constraints from spatial probability maps derived from U-Net, a deep learning model, and Hessian convexity maps derived from Hessian analysis on the DoG detector. The theoretical foundation of Hessian analysis guarantees that pre-segmentation will recognize all true convex blobs and some non-blob convex objects, resulting in a blob superset. Joining probability maps allows us to distinguish true blobs from the superset. The joint-constraint extension of the detector requires no post-pruning and thus is robust, generalizable and computationally efficient.

Within the field of deep learning, the Convolutional Neural Network (CNN) has been successfully implemented in medical imaging applications ranging from object detection and segmentation to classification^35–37^. The first generation of CNN models was used to classify images through fully connected layers. Shelhamer *et al*.^38^ first proposed a Fully Convolutional Network (FCN) that transfers the fully connected layers to deconvolutional layers and provides a dense class map with arbitrarily-sized input image. The FCN changes “image-label” mapping to “pixel/voxel-label” mapping for object detection and image segmentation. One limitation of the FCN for medical imaging is the need for large training datasets. A lightly weighted FCN model, the U-Net^39^, employs a modified FCN architecture to require fewer training images but yield precise, fast segmentation. U-Net has been implemented in various medical segmentation tasks such as nucleus, cell, and breast lesion segmentation^40–42^, all drawn from limited datasets. The U-Net yields a probability map where each pixel or voxel indicates the likelihood of being within the imaging object. However, based on our previous study^43^, U-Net does not reliably separate glomeruli within close proximity. Therefore, we choose to adopt the probability map as part of UH-DoG in conjunction with Hessian analysis for glomerulus detection from CFE-MR images.

There are three main advantages of the UH-DoG method. First, a global blob likelihood constraint from the U-Net probability map reduces over-detection by DoG. Second, a local convex constraint from the Hessian convexity map reduces under-segmentation. Third, integrating the probability map constraint with the Hessian convexity map eliminates the need for post-pruning. To validate the performance of UH-DoG, four methods were chosen from the literature: HLoG^1^, gLoG^21^, LoG^22^, and Radial-Symmetry^17^. We tested these on dataset of 2D fluorescent images (n = 200) where the locations of blobs were known. UH-DoG outperformed the other four methods in F-score and performed comparably to the other four methods in recall. Next, we compared blob detection of these methods on a 3D kidney MR dataset against the HDoG method. The differences between UH-DoG and HDoG were negligible but the average computation time of UH-DoG was 35% shorter than that of HDoG.

## Methods

**Figure 1:**
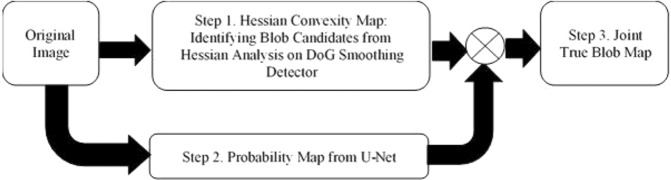
Proposed UH-DoG for glomerulus identification.

We propose UH-DoG, a joint constraint-based detector for glomeruli detection. UH-DoG consists of three steps (Fig. 1). Step 1 is to use the Difference of Gaussian (DoG) to smooth the images, followed by Hessian analysis to identify possible blob candidates based on local convexity. Step 2 is to use a trained U-Net to generate a probability map, which captures the most likely blob locations. Step 3 is to combine the probability map from Step 2 with blob candidates from Step 1 as joint constraints to identify true blobs. Each step is discussed in detail in the following sections.

### Hessian analysis and hessian convexity map

Before implementing Hessian analysis, DoG is used to smooth the images. By employing a convolution operator, DoG can filter image noise and enhance objects at the selected scale^24^. DoG is a fast approximation of the LoG filter to highlight blob structure^4^ and is thus computationally efficient^18^.

Let a 3D image be $f:{R}^{3}\to R.\,$The scale-space representation $L(x,y,z;\sigma )$ at point (*x*, *y*, *z*), with scale parameter *σ*, is the convolution of image *f*(*x*, *y*, *z*) with the Gaussian kernel $G(x,y,z;\sigma )$:

1 $$L(x,y,z;\sigma )=\,G(x,y,z;\sigma )\ast f(x,y,z),$$

where * is the convolution operator and the Gaussian kernel $G(x,y,z;\sigma )=\,\frac{1}{{(2\pi {\sigma }^{2})}^{\frac{3}{2}}}{e}^{-\frac{({x}^{2}+{y}^{2}+{z}^{2})}{2{\sigma }^{2}}}$. The Laplacian of $L(x,y,z;\sigma )$ is:

2 $${\nabla }^{2}L(x,y,z;\sigma )=\frac{{\partial }^{2}L(x,y,z;\sigma )}{\partial {x}^{2}}+\frac{{\partial }^{2}L(x,y,z;\sigma )}{\partial {y}^{2}}+\frac{{\partial }^{2}L(x,y,z;\sigma )}{\partial {z}^{2}}.$$

According to^18^, ${\rm{\sigma }}{\nabla }^{2}L(x,y,z;\sigma )={L}_{\sigma }(x,y,z;\sigma ).\,$We approximate the partial derivative ${L}_{\sigma }(x,y,z;\sigma )$ by a one-sided difference quotient, the DoG approximation of LoG is:

3 $$\begin{array}{rcl}{\nabla }^{2}L(x,y,z;\sigma ) & = & \frac{{L}_{\sigma }(x,y,z;\sigma )\,}{{\rm{\sigma }}}\approx \frac{L(x,y,z;\sigma +\Delta \sigma )-L(x,y,z;\sigma )}{{\rm{\sigma }}\Delta \sigma }\\  & = & f(x,y,z)\ast \frac{(G(x,y,z;\sigma +\Delta \sigma )-G(x,y,z;\sigma ))}{{\rm{\sigma }}\Delta \sigma }\end{array}$$

To locate an optimum scale for the blobs, similar to^1^, we add γ-normalization to form the normalized DoG detector ${\sigma }^{\gamma }{\nabla }^{2}L(x,y,z;\sigma )$, which is:

4 $$Do{G}_{nor}(x,y,z;\sigma )={\sigma }^{\gamma -1}f(x,y,z)\ast \frac{(G(x,y,z;\sigma +\Delta \sigma )-G(x,y,z;\sigma ))}{\Delta \sigma },$$

where γis introduced to automatically determine the optimum scale for the blobs. We set *γ* to 2 here. For details on tuning *γ*, refer to^1^. The normalized DoG transformation underlies Hessian-based convexity analysis to detect blobs.

After the image is smoothed by the normalized DoG, for a voxel (*x*, *y*, *z*) in the normalized DoG image $Do{G}_{nor}(x,y,z;\sigma )$ at scale *σ*, the Hessian matrix for this voxel is:

5 $$H(Do{G}_{nor}(x,y,z;\sigma ))=[\begin{array}{ccc}\frac{{\partial }^{2}Do{G}_{nor}(x,y,z;\sigma )}{\partial {x}^{2}} & \frac{{\partial }^{2}Do{G}_{nor}(x,y,z;\sigma )}{\partial x\partial y} & \frac{{\partial }^{2}Do{G}_{nor}(x,y,z;\sigma )}{\partial x\partial z}\\ \frac{{\partial }^{2}Do{G}_{nor}(x,y,z;\sigma )}{\partial x\partial y} & \frac{{\partial }^{2}Do{G}_{nor}(x,y,z;\sigma )}{\partial {y}^{2}} & \frac{{\partial }^{2}Do{G}_{nor}(x,y,z;\sigma )}{\partial y\partial z}\\ \frac{{\partial }^{2}Do{G}_{nor}(x,y,z;\sigma )}{\partial x\partial z} & \frac{{\partial }^{2}Do{G}_{nor}(x,y,z;\sigma )}{\partial y\partial z} & \frac{{\partial }^{2}Do{G}_{nor}(x,y,z;\sigma )}{\partial {z}^{2}}\end{array}].$$

In a normalized DoG-transformed 3D image, each voxel of a transformed bright blob has a negative definite Hessian matrix^28^. We define a binary indicator matrix, $HI(x,y,z;\sigma )$, termed the Hessian convexity map. $HI(x,y,z;\sigma )=1$ when $H(Do{G}_{nor}(x,y,z;\sigma ))$ is negative definite; otherwise, $HI(x,y,z;\sigma )=0$.

To determine a single optimum scale σ^*^, the maximum value of the normalized DoG is used here^28^. Let the average DoG value per blob candidate voxel measure *B*_*GoG*_ be:

6 $${B}_{DoG}(\sigma )=\frac{{\sum }_{(x,y,z)}DoG(x,y,z)HI(x,y,z;\sigma )}{{\sum }_{(x,y,z)}HI(x,y,z;\sigma )}.$$

We have ${\sigma }^{\ast }=argmax\,{B}_{DoG}(\sigma )$. σ^*^ is used to generate the optimum Hessian convexity map $HI(x,y,z;{\sigma }^{\ast })$. This map is the local convexity constraint for detecting the convex blob regions. Result is a set of convex objects including all true blobs and some non-blob convex objects.

### U-Net and Probability Map

A classical CNN usually consists of multiple convolutional layers followed by pooling layers, activation layers, and fully connected layers. Convolutional layers learn hierarchical and high-level feature representation. Pooling layers can reduce feature dimensions and capture spatial feature invariance. The final fully-connected layers categorize the images into different groups. Compared to classical CNNs, FCNs replace all fully-connected layers with a fully convolutional layer. There are several advantages to this approach. First, the input image size can be arbitrary because all models consist of convolution layers, so output size only depends on input size. Second, the FCN can be trained from whole images without patch sampling, thus the effects of patch-wise training need not be considered. However, FCNs require a large dataset for training. To address this issue, a modified FCN model, the U-Net, was proposed. Interested readers are referred to U-Net^39^ for details. The output of U-Net is a probability map in [0, 1].

In U-Net, let the input images be $X\in {R}^{{I}_{1}\times {I}_{2}\times {I}_{3}}$, and the output map be $Y\in {R}^{{I}_{1}\times {I}_{2}\times {I}_{3}}$, where ${y}^{{i}_{1},{i}_{2},{i}_{3}}\in [0,1]$. A binary cross entropy loss function is used in the training process to obtain the output map:

$$L=-\,\frac{1}{{I}_{1}{I}_{2}{I}_{3}}\mathop{\sum }\limits_{k=1}^{{I}_{1}\times {I}_{2}\times {I}_{3}}{Y}_{k}\Delta \,\log ({\hat{Y}}_{k})+(1-{Y}_{k})\Delta \,\log (1-{\hat{Y}}_{k}),$$

where *Y*_*k*_ is the true label and ${\hat{Y}}_{k}\,$is the predicted probability for voxel k.

In our U-Net output, we obtain a probability map $U(x,y,z),\,$where *x* and *y* are dimensions of each 2D image slice and *z* is the slice number. The probability map is the global blob likelihood constraint in our joint constraints operations to detect the most likely blob regions. By setting a probability threshold, most noise is removed. However, some touching blobs could have a higher probability than the threshold in the boundary and might not be split up, resulting in reduced detection known as under-segmentation. Joining the Hessian convexity map with U-Net probability map will address the challenges of small blob detection.

### Joint constraint operation for true blob identification

Given a 3D image $f:{R}^{3}\to R,$ Hessian analysis is applied to render a convexity map $\,HI(x,y,z;{\sigma }^{\ast })$, U-Net is applied to render a probability map $U(x,y,z)$. We introduce a joint operator

7 $$UH(x,y,z)=HI(x,y,z;{\sigma }^{\ast })\circ I(x,y,z),$$

where $I(x,y,z)$ is a binary indicator matrix. Given a probability threshold ${\delta }_{b},\,I(x,y,z)$ = 1 when $U(x,y,z) > {\delta }_{b}$; otherwise, $I(x,y,z)$ = 0. We define the true blob candidate as a 27-connected voxel^44^, and the blob set is represented as:

8 $${S}_{blob}=\{(x,y,z)|(x,y,z)\in Do{G}_{nor}(x,y,z;\sigma ),UH(x,y,z)=1\,\}.$$

**Figure 2:**
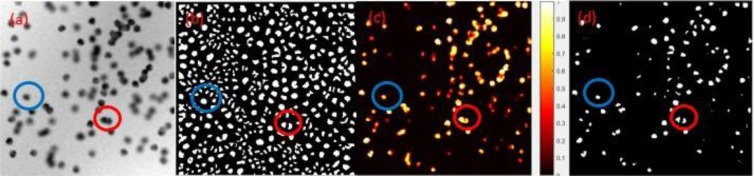
(**a**) A 2D gray scale image preprocessed from experiment 1 fluorescent image (**b**) Binary Hessian convexity map of (**a**), the convex pixels are marked as the white color. (**c**) U-Net probability map of (**a**), pixel is illustrated with a color indicating a probability of the pixel belonging to a blob. (**d**) Blob identification map joined from Hessian convexity map and U-Net probability map with 0.5 threshold.

To illustrate, Fig. 2 shows images of blobs detected during the joint constraint operation of the U-Net probability map and the Hessian convexity map. The blue circle in Fig. 2a shows only one blob. The same blue circle on Fig. 2b, after application of the Hessian convexity map, shows there is one “bigger” blob in the middle and a number of smaller blobs around the boundary of the blue circle. Figure 2c shows the correct outcome, only one blob in the middle. This clearly illustrates the sensitivity of the Hessian matrix to noise. Even though the Hessian analysis guarantees the detection of the convex object, some non-blob convex objects (noise) will also be detected, resulting in over-detection. This noise can be readily filtered by the U-Net probability map (Fig. 2c). We conclude that U-Net may be useful for denoising, which alleviates the over-detection of Hessian analysis.

The red circle in Fig. 2a shows overlapped blobs. They are still overlapping in the U-Net probability map from Fig. 2c. But they are split up in the Hessian convexity map from Fig. 2b. By joining the Hessian convexity map and U-Net probability map with a single global threshold, the overlapped blobs in the red circle are visualized as distinct entities, as shown in Fig. 2d. We conclude Hessian analysis could alleviate the under-segmentation issue from U-Net.

Our proposed UH-DoG integrates the probability map from U-Net and convexity map from Hessian analysis to guarantee robustness to noise and effective blob detection. The detailed steps of UH-DoG are shown in Table 1.

**Table 1:** Detail Steps of proposed UH-DoG.

1. Use a pretrained model to generate a probability map of blobs from original image
2. Initialize the normalization factor *γ*, and range and step-size of parameter *σ*, to transform the original image into normalized DoG space.
3. Calculate the Hessian matrix based on normalized DoG smoothed image and generate the Hessian convexity map $HI(x,y,z;\sigma )$.
4. Calculate average DoG intensity ${B}_{DoG}(\sigma )=\frac{{\sum }_{(x,y,z)}DoG(x,y,z)HI(x,y,z;\sigma )}{{\sum }_{(x,y,z)}HI(x,y,z;\sigma )}$ and find the optimum scale section by$\,{\sigma }^{\ast }=argmax\,{B}_{DoG}(\sigma )$.
5. Get the optimum Hessian convexity map $HI(x,y,z;{\sigma }^{\ast })$ under scale σ^*^.
6. Join the probability map with Hessian convexity map to identify true blobs.

### Experiments

Two experiments were conducted to validate of the performance of our proposed UH-DoG detector. The first experiment validated the UH-DoG on 200 fluorescence, 2D light microscopy images for cell detection^45^. The 2D cell images were of interest because (1) to the best of our knowledge, there are no 3D small blob datasets available for comparison; (2) the blobs from these images are small and each image could be used to test the performance of the algorithm in the presence of background noise; (3) this dataset has the ground truth of the locations of each blob. The detection accuracy measured by recall, precision, and F-score can be used to compare this approach with methods from the literature. The second experiment validated the performance of UH-DoG on CFE-MR images of mouse kidneys where each glomerulus was detected. All experiments were approved by the University of Virginia Institutional Care and Use Committee, in accordance with the NIH Guide for the Care and Use of Laboratory Animals.

## Results

### Training dataset and data augmentation

**Figure 3:**
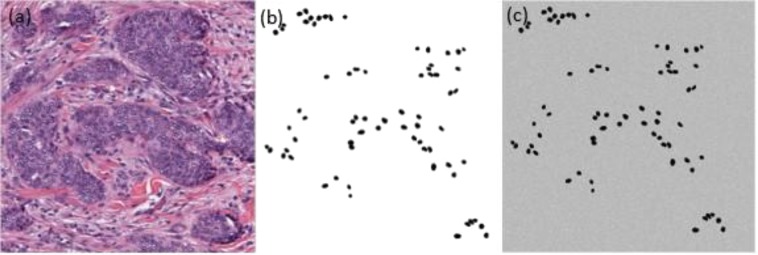
Training images. (**a**) Original image. (**b**) Ground truth labeled image. (**c**) Simulated training image.

We used a public dataset^46^ to train our deep learning model, based on optical images of cell nuclei. This dataset has 141 optical microscopy pathology images (2,000 × 2,000 pixels), as shown in Fig. 3a. The 12,000 ground truth annotations are typically done by an expert, which involves delineating object boundaries over 40 hours^46^. Due to the large amount of time and effort required, the annotated nuclei in this dataset only represents a small fraction of the total number of nuclei present in all images. Since we aim to facilitate U-Net to denoise our blobs images based on the ground truth labeled images, as shown in Fig. 3b, we generated Gaussian distributed noise with ${\mu }_{noise}=0$ and ${\sigma }_{noise}^{2}=0.01$ and we added it to the ground truth labeled images, resulting in 141 simulated training images, as shown in Fig. 3c. Data were augmented to increase the in variance and robustness properties of U-Net^39^. We generated the augmented data by a combination of rotation shift, width shift, height shift, shear, zoom, and horizontal flip.

### Experiment I: Validation experiments using 2D fluorescent images

**Figure 4:**
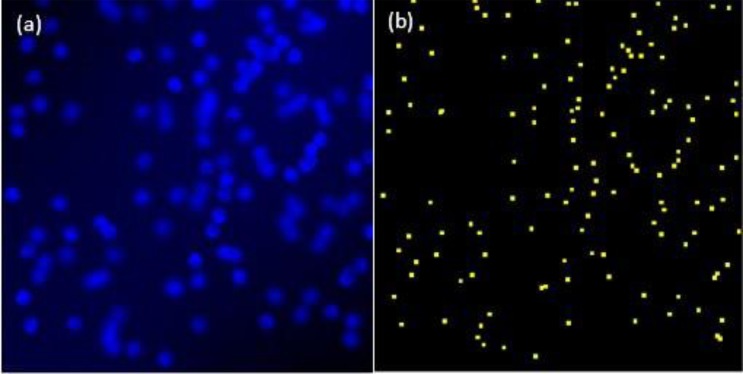
(**a**) Sample 2D fluorescent image. (**b**) Ground truth dots of (**a**).

Figure 4 illustrates an example fluorescent image (256 × 256 pixels). Since this was a 2D image, our proposed UH-DoG must incorporate a modified 2D DoG because comparison algorithms were from the 2D LoG and its extensions.

To revise the DoG to a 2D version, for 2D images $f(x,y)$ with the Gaussian kernel $G(x,y;\sigma )$, we modified the normalized 3D DoG detector from Eq. 4 in a 2D format:

9 $$Do{G}_{nor}(x,y;\sigma )={\sigma }^{\gamma -1}f(x,y)\ast \frac{(G(x,y;\sigma +\Delta \sigma )-G(x,y;\sigma ))}{\sigma \Delta \sigma }.$$

Then the corresponding Hessian matrix were modified from Eq. 5 as follows:

10 $$H(Do{G}_{nor}(x,y;\sigma ))=[\begin{array}{cc}\frac{{\partial }^{2}Do{G}_{nor}(x,y;\sigma )}{\partial {x}^{2}} & \frac{{\partial }^{2}Do{G}_{nor}(x,y;\sigma )}{\partial x\partial y}\\ \frac{{\partial }^{2}Do{G}_{nor}(x,y;\sigma )}{\partial x\partial y} & \frac{{\partial }^{2}Do{G}_{nor}(x,y;\sigma )}{\partial {y}^{2}}\end{array}].$$

The parameter settings for Hessian analysis and DoG were as suggested in^28^. *γ* is set to 2. *σ* varies from 0.5 to 3 with step-size 0.5. Δ*σ* is set to 0.001.

We used precision, recall and F-score to evaluate the performance of our proposed algorithm. Precision measures the fraction of retrieved candidates confirmed by the ground-truth. Recall measures the fraction of ground-truth data retrieved. F-score measures overall performance. Since ground truth data were provided in the form of dots (the coordinates of the blob centers), as in the literature^1,28^, a candidate was considered a true positive if its intensity centroid was within a threshold *d* of the corresponding ground truth dot. Specifically, if the Euclidian distance *D*_*ij*_ between dot *i* and blob candidate j was less than or equal to *d*, the blob was considered a true positive. To avoid duplicate counting, the number (#) of true positives *TP* was calculated by Eq. 11. Precision, recall, and F-score are calculated by Eqs. 12, 13, 14 respectively:

11 $$TP=\,\min \{\#\{j:{\min }_{i=1}^{m}{D}_{ij}\le d\},\,\#\{i:{\min }_{j=1}^{n}{D}_{ij}\le d\}\},$$

12 $$precision=\frac{TP}{n},$$

13 $$recall=\frac{TP}{m}$$

14 $$F-score=2\times \frac{precision\times recall}{(precision+recall)},$$

where *m* is the number of ground-truth and *n* is the number of blob candidates; *d* is a thresholding parameter set to a positive value $\,(0,+\infty )$. If *d* is small, fewer blob candidates are counted since the distance between the blob candidate centroid and ground-truth should be small. If *d* is too large, more blob candidates are counted. Here, since local intensity extremes could be anywhere within a small blob with an irregular shape, we set *d* to the average diameter of the blobs: $d=2\times \sqrt{\frac{{\sum }_{(x,y)}I(x,y;\sigma )}{\pi }}$.

Since the results of detection by the complete versions of HLoG, gLoG, Radial-Symmetry and LoG on 200 pathological images are available online^1,17,21^, the results were directly used from these papers for comparison.

**Figure 5:**
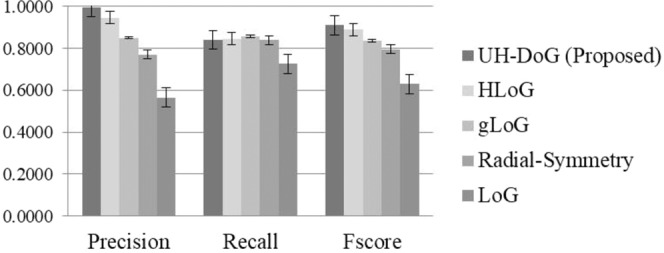
Comparison of full versions of UH-DoG, HLoG, gLoG, Radial-Symmetry and LoG on 200 fluorescence images. The error bar indicates the standard deviation of the corresponding measure across 200 images. For precision and F-score, UH-DoG has significant different (see Table 2) with others. For recall, UH-DoG has significant difference with gLoG and LoG.

Figure 5 shows a comparison of UH-DoG to the HLoG, gLoG, LoG and Radial-Symmetry algorithms. While UH-DoG is comparable to HLoG, gLoG and Radial Symmetry algorithms in recall, it significantly outperforms the four algorithms in both precision and F-score (Table 2). The standard deviation of F-score in UH-DoG was 0.025, compared to 0.0377 with the HLoG method, compared to 0.1436 with the gLoG method, 0.0795 with the Radial-Symmetry method, and 0.0385 with the LoG method. We conclude that UH-DoG provides more accurate and robust detection of blobs in this dataset. In addition, statistical analysis was performed with the results summarized in Table 2. While comparable to the four algorithms on recall, our approach statistically outperformed the others on precision and F-score.

**Table 2:** ANOVA using Tukey’s HSD pairwise test on 200 Fluorescent Images (*significance *p* < 0.05).

UH-DoG vs.	Precision	Recall	F-Score
HLoG	*<**0**.**0001**	0.207	*<**0**.**0001**
gLoG	*<**0**.**0001**	*0.**001**	*<**0**.**0001**
Radial Symmetry	*<**0**.**0001**	0.963	*<**0**.**0001**
LoG	*<**0**.**0001**	*<**0**.**0001**	*<**0**.**0001**

### Experiment II: Validation experiments using 3D Kidney MRI

In this section, we conducted experiments on CF-labeled glomeruli from a dataset of 3D magnetic resonance images (256 × 256 × 256 voxels) to measure number (N_glom_) and apparent size (aV_glom_) of glomeruli in diseased kidneys and healthy control kidneys. Acute kidney injury was induced in adult male C57Bl/6 mice using an intraperitoneal injection of folic acid (125 mg). A subset of the group receiving folic acid, the AKI group (n = 4) was euthanized 4 days after the folic acid was administered and the remainder of those that received folate were euthanized 4 weeks later and termed the chronic kidney disease (CKD) group, n = 3. The control groups for AKI (n = 5) and CKD (n = 6) were age-matched adult male C57Bl/6 mice that received intraperitoneal sodium bicarbonate.

**Figure 6:**
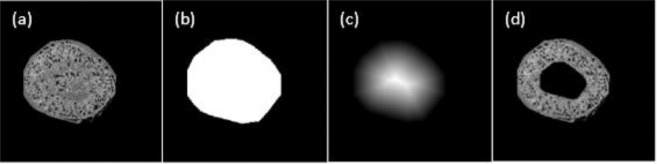
(**a**) One slice of healthy mouse kidney (ID: 477) image. (**b**) Binary image of (**a**). (**c**) Distance mask of (**b**). (**d**) Remove medulla from (**a**).

For improved detection, we adopted a preprocessing step to segment the medulla from the image because no glomeruli are located there. Based on the segmented kidney image, shown in Fig. 6a, we converted it to a binary mask (Fig. 6b). Then we generated a distance mask, seen in Fig. 6c. With the map showing the distance between each kidney’s voxel and the kidney boundary, we set up a distance threshold to remove regions farther from the boundary than this threshold. Figure 6d shows the 2D image slice after removing the medulla.

**Figure 7:**
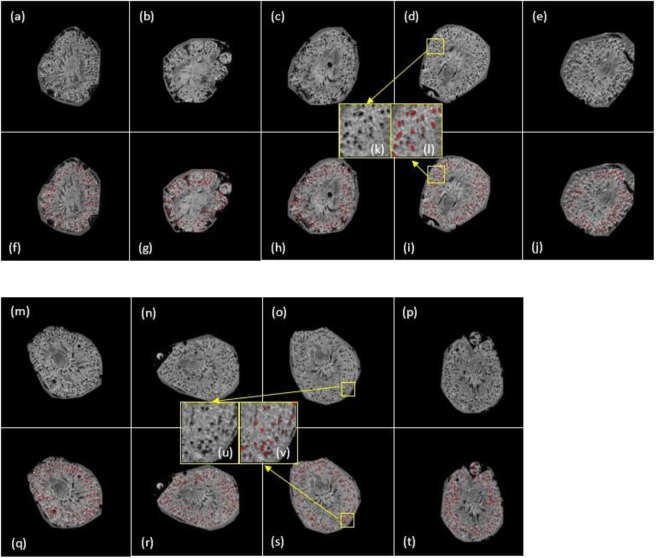
(**a**) Glomerular segmentation results from 3D MR images of mouse kidneys (selected slices presented). (**a**–**e**) One slice for the CKD group. (**f**–**j**) Identified glomeruli are marked in red. (**k**) is the zoom-in region of (**d**) while (**l**) is the segmentation result of (**k**). (**b**) Glomerular segmentation results from 3D MR images of mouse kidneys (selected slices presented). (**m**–**p**) One slice for the control group. (**q**–**t**) Identified glomeruli are marked in red. (u) is the zoom-in region of (o) while (v) is the segmentation results of (u).

**Figure 8:**
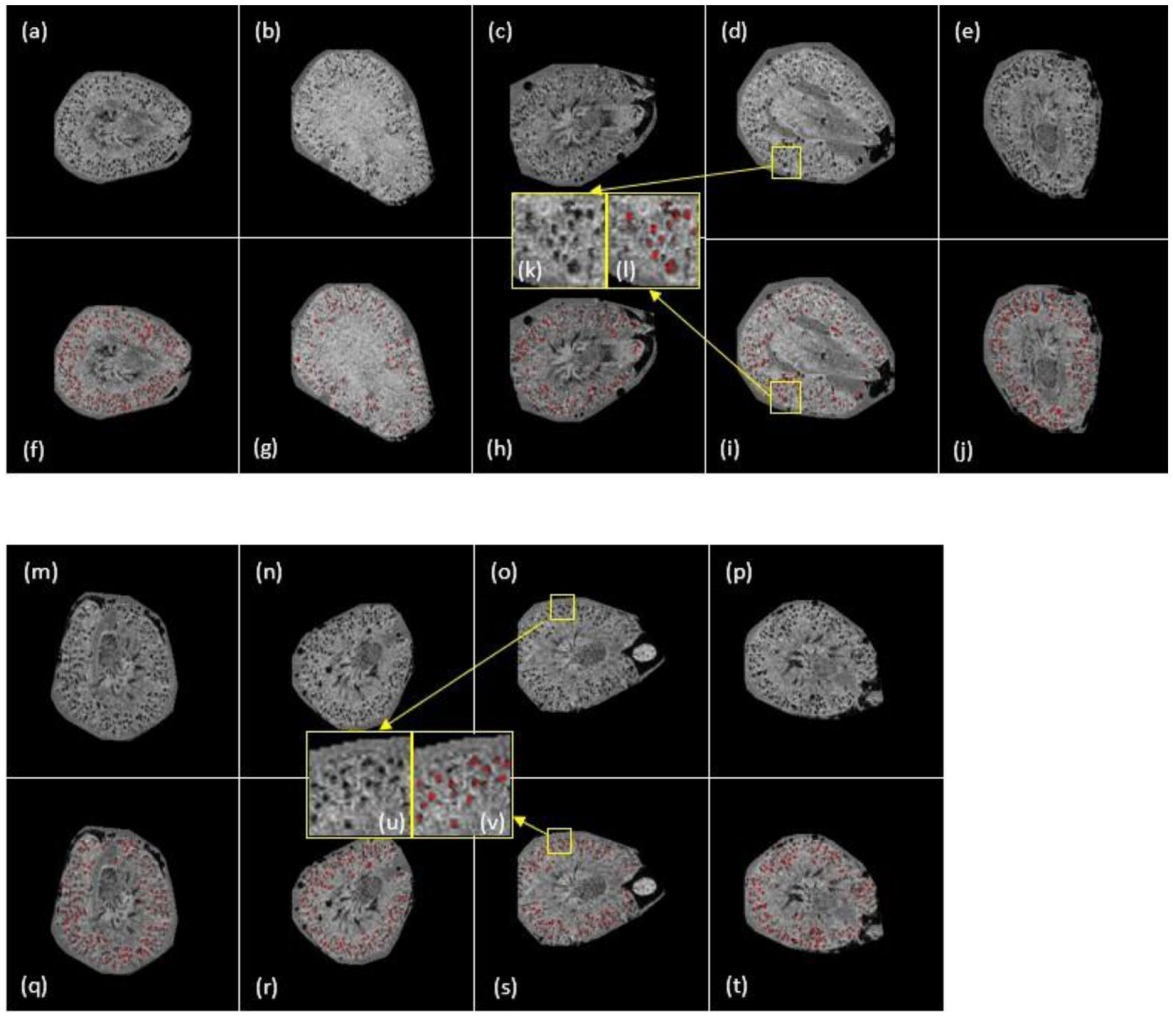
(**a**) Glomerular segmentation results from 3D MR images of mouse kidneys (selected slices presented). (**a**–**e**) One slice for the AKI group. (**f**–**j**) Identified glomeruli are marked in red. (k) is the zoom-in region of (**d**) while (**l**) is the segmentation result of (**k**). (**b**) Glomerular segmentation results from 3D MR images of mouse kidneys (selected slices presented). (**m–p**) One slice for the control group. (**q–t**) Identified glomeruli are marked in red. (u) is the zoom-in region of (o) while (v) is the segmentation results of (u).

Then we performed the proposed UH-DoG method to segment the kidney glomeruli in Fig. 6d. The parameter settings are as follows: *γ* is set to 2. *σ* varies from 0.5 to 1.8 with step-size 0.1. Δ*σ* is set to 0.001. Example segmentation results are shown in Figs. 7 and 8. The number of glomeruli (N_glom_), mean apparent glomerular volume (aV_glom_) and median aV_glom_ are reported in Table 3, where the UH-DoG method is compared to the HDoG method. We used the method of calculating apparent glomerular volume from the paper^34^. Similarly, Table 4 summarizes the results from the AKI and control groups.

**Table 3:** Glomerular number (Nglom) and volume (aVglom) for the CKD and control mice kidneys using the proposed UH-DoG method comparing with HDoG method (*aVglom unit *mm*^3^ × 10^−4^).

Mouse		N_glom_ (UH-DoG)	N_glom_ (HDoG)	N_glom_ Difference Ratio (%)	Mean aV_glom_ (UH-DoG)	Mean aV_glom_ (HDoG)	Mean aV_glom_ Difference Ratio (%)	Median aV_glom_ (UH-DoG)	Median aV_glom_ (HDoG)	Median aV_glom_ Difference Ratio (%)
CKD	ID 429	7,346	7,656	**4**.**05**	2.92	2.57	**11**.**99**	1.74	1.48	**14**.**94**
	ID 466	8,138	8,665	**6**.**08**	2.06	2.01	**2**.**43**	1.15	0.94	**18**.**26**
	ID 467	8,663	8,549	**1**.**33**	2.32	2.16	**6**.**90**	1.47	1.28	**12**.**93**
	Avg	8,049	8,290	**2**.**91**	2.43	2.25	**7**.**67**	1.45	1.23	**15**.**14**
	Std	663	552		0.44	0.29		0.30	0.27	
Control	ID 427	12,701	12,724	**0**.**18**	1.61	1.49	**7**.**45**	1.26	1.15	**8**.**73**
	ID 469	11,347	10,829	**4**.**78**	2.20	1.91	**13**.**18**	1.41	1.20	**14**.**89**
	ID 470	11,309	10,704	**5**.**65**	2.04	1.98	**2**.**94**	1.50	1.37	**8**.**67**
	ID 471	12,279	11,943	**2**.**81**	1.56	1.5	**3**.**85**	1.22	1.13	**7**.**38**
	ID 472	12,526	12,569	**0**.**34**	1.49	1.35	**9**.**40**	1.16	1.06	**8**.**62**
	ID 473	11,853	12,245	**3**.**20**	1.58	1.50	**5**.**06**	1.25	1.18	**5**.**60**
	Avg	12,003	11,836	**1**.**41**	1.75	1.62	**7**.**16**	1.30	1.18	**9**.**10**
	Std	595	872		0.30	0.26		0.13	0.10

**Table 4:** Glomerular number (Nglom) and volume (aVglom) for the AKI and control mice kidneys using the proposed UH-DoG method comparing with HDoG method (*aVglom unit *mm*^3^ × 10^−4^).

Mouse		N_glom_ (UH-DoG)	N_glom_ (HDoG)	N_glom_ Difference Ratio (%)	Mean aV_glom_ (UH-DoG)	Mean aV_glom_ (HDoG)	Mean aV_glom_ Difference Ratio (%)	Median aV_glom_ (UH-DoG)	Median aV_glom_ (HDoG)	Median aV_glom_ Difference Ratio (%)
AKI	ID 433	11,033	11,046	**0**.**12**	1.63	1.53	**6**.**13**	1.27	1.17	**7**.**87**
	ID 462	10,779	11,292	**4**.**54**	1.48	1.34	**9**.**46**	1.17	1.00	**14**.**53**
	ID 463	10,873	11,542	**5**.**80**	2.61	2.35	**9**.**96**	1.60	1.25	**21**.**88**
	ID 464	11,340	11,906	**4**.**75**	2.40	2.31	**3**.**75**	1.59	1.17	**26**.**42**
	Avg	11,006	11,447	**3**.**85**	2.03	1.88	**7**.**27**	1.41	1.15	**18**.**47**
	Std	246	367		0.56	0.52		0.22	0.11	
Control	ID 465	10,115	10,336	**2**.**14**	2.40	2.30	**4**.**17**	1.66	1.42	**14**.**46**
	ID 474	11,157	10,874	**2**.**60**	2.52	2.44	**3**.**17**	1.70	1.44	**15**.**29**
	ID 475	10,132	10,292	**1**.**55**	1.70	1.74	**2**.**35**	1.26	1.16	**7**.**94**
	ID 476	10,892	10,954	**0**.**57**	1.62	1.53	**5**.**56**	1.21	1.09	**9**.**92**
	ID 477	11,335	10,885	**4**.**13**	1.70	1.67	**1**.**76**	1.27	1.19	**6**.**30**
	Avg	10,726	10,668	**0**.**54**	1.99	1.94	**2**.**62**	1.42	1.26	**11**.**27**
	Std	572	325		0.43	0.41		0.24	0.16	

We performed quality control by visually checking the identified glomeruli in kidney images. Figure 7 shows glomerular identification for CKD and control kidneys. Figure 8 shows glomerular identification for kidneys in the AKI and control groups.

### Discussion: computation cost

UH-DoG significantly decreases computation time compared to the HDoG algorithm^28^, as shown in Tables 5 and 6. The training time of U-Net is not included in the estimates of computation time as it is trained beforehand and can be used to test on all images.

**Table 5:** Computation time for CKD and Control kidneys using HDoG and the proposed method with scale = 1 (Intel Xeon 3.6 GHz CPU and 16 GB of memory, NVIDIA TITAN XP and 12 GB of memory).

Mouse		HDoG (seconds)	UH-DoG (seconds)
CKD	ID 429	9.3	7.3
	ID 466	9.5	7.3
	ID 467	11.4	7.6
	Avg	10.1	7.4
	Std	1.2	0.2
Control	ID 427	11.7	8.2
	ID 469	11.7	8.0
	ID 470	12.0	8.0
	ID 471	11.9	8.0
	ID 472	12.0	8.1
	ID 473	25.2	8.2
	Avg	14.1	8.1
	Std	5.5	0.1

**Table 6:** Computation time for AKI and Control kidneys using HDoG and the proposed method with scale = 1 (Intel Xeon 3.6 GHz CPU and 16 GB of memory, NVIDIA TITAN XP and 12 GB of memory).

Mouse		HDoG (seconds)	UH-DoG (seconds)
AKI	ID 433	13.7	7.9
	ID 462	13.4	8.0
	ID 463	13.1	8.0
	ID 464	14.3	8.3
	Avg	13.6	8.1
	Std	0.5	0.2
Control	ID 465	11.0	7.8
	ID 474	12.3	8.0
	ID 475	11.4	7.8
	ID 476	12.0	8.1
	ID 477	11.6	7.9
	Avg	11.7	7.9
	Std	0.5	0.1

### Discussion: Clinical translation

The use of imaging biomarkers in humans has increased both for disease early detection and disease severity assessment. Additionally, imaging biomarkers can serve as surrogate endpoints in clinical trials, reducing cost and burden associated with these studies. For example, total kidney volume has recently been accepted as a surrogate marker for disease progression in autosomal dominant polycystic kidney disease trials^47^. Although the importance of glomerular number has been universally accepted, the detection of glomerular number and size has been limited because the only methodology to obtain these metrics were destructive stereological approaches that could only be performed post mortem. With the advent of CFE-MRI, the need for image analysis tools is paramount. However, it is critical to the success of any imaging biomarker that the marker be accurate and rapidly obtained. This study demonstrates some of the challenges in detecting small objects, such as glomeruli, particularly in the settings of low image resolution, image noise and overlap of objects. It also shows the promise of rapid acquisition where data can be used in a timeframe to influence patient care. Further work is necessary to validate the accuracy of the detection of diseased glomeruli to apply this algorithm to a wider range of renal disease models.

## Conclusion

Discovering imaging biomarkers is important to inform disease diagnosis, prognosis, therapy development and treatment assessment. Of particular interest in this research is to identify quantitative glomeruli biomarkers from CFE-MR image. This is a challenging problem because the number of glomeruli is large, the size is small. In addition, the limitation from imaging acquisition such as hardware and variable acquisition parameters often renders the images with less desirable resolution resulting the overlapping glomeruli. In this paper, we demonstrated a new small blob detector by joining the Hessian convexity map and probability map from U-Net. This joint constraint-based approach overcomes under-segmentation by U-Net and over-detection by Hessian analysis. While it was successfully implemented in segmenting the kidney glomeruli, there are still some limitations. First, the assumption that the blobs are convex and similar in size may not be robust for non-convex objects with difference sizes. A future possible improvement is to enhance ability of U-Net to detect both convex and non-convex small objects. Second, the probability map is sensitive to the threshold. We plan to explore the use of thresholding to improve UH-DoG.

## Data Availability

The datasets generated during and/or analyzed during the current study will be made available upon request.
